# Non‐scheduled short‐acting opioid to taper off opioids?

**DOI:** 10.1111/cns.14705

**Published:** 2024-04-07

**Authors:** Renyu Liu, Bonnie Milas, John Grothusen

**Affiliations:** ^1^ Department of Anesthesiology and Critical Care Perelman School of Medicine at the University of Pennsylvania Philadelphia Pennsylvania USA; ^2^ Department of Neurology Perelman School of Medicine at the University of Pennsylvania Philadelphia Pennsylvania USA

**Keywords:** addiction, dezocine, long‐acting, opioids, short‐acting, therapy

## Abstract

This commentary discusses the issues related to the current pharmacotherapy using super long‐acting opioids (for the potential convenience for both patients and medical providers) for opioid addiction and argues for the potential to use a non‐scheduled short‐acting opioid to taper off opioids to reduce total number of patients on opioids and ultimately reduce opioid‐related death. This article also proposes to develop short‐acting opioids for addiction management instead of the current long‐acting regimen. The authors further suggest that dezocine, a previously FDA approved medication for perioperative pain management and a non‐scheduled opioid, be brought back to clinical practice in the US as a potential alternative addiction management medication, especially for those who are highly motivated to quit opioids completely using a taper off strategy.

## Graphical abstract

In this perspective, we are proposing to introduce a non‐scheduled short‐acting opioid into clinical practice given the significant need to find alternatives to taper off opioids and bridge buprenorphine and methadone therapies for opioid use disorders. Dezocine, a previously FDA approved non‐scheduled opioid for pain management, is a potential candidate to explore. Dr. Renyu Liu obtained the images presented in this graphic abstract from Adobe Stock with standard licenses.

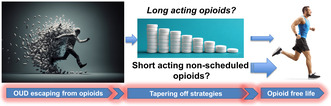

Opioid use disorder (OUD) affects over 16 million people worldwide, with more than 2.1 million in the United States alone, resulting in over 120,000 deaths annually worldwide.^1^ Although the drugs causing overdose are moving towards shorter acting opioids like fentanyl, “therapeutic medications” are moving towards more long‐acting ones in various formulations with active metabolites.

The market for medical treatment of opioid use disorder (OUD) is growing rapidly every year. From 2015 to 2022, the total spent on medications to treat OUD in the US alone increased from $1.2 billion to $2.4 billion, with naltrexone increasing from $139 million to $550 million, methadone from $324 million to $557 million, and buprenorphine from $779 million to $1.2 billion. By 2026 the total cost in the US for medical treatment of OUD is projected to be over $3.5 billion, made up of naltrexone ($985 million), methadone ($771 million) and buprenorphine ($1.7 billion).^2^ Buprenorphine is becoming the main medication for medical treatment of OUD. The global buprenorphine market in 2019 was about $3.7 billion and is projected to rise to $10.9 billion by the year 2027 even though buprenorphine is an old drug and the patent on the molecule has long expired. The huge market demands have attracted more than 13 pharmaceutical companies to produce buprenorphine in various patented formulations to prolong the pharmacological effect of long‐acting buprenorphine.^3^ Suboxone, a combination of naloxone and buprenorphine, has half‐life time of 24–36 h and its metabolite, norbuprenorphine, can be detected in the blood or urine after 14 days. Sublocade, a subcutaneous extended‐release form, can maintain the “therapeutic level” for 1 month; Probuphine, a subdermal implant (now discontinued), can last 6 months; Subutex, a sublingual tablet, can have detectable buprenorphine in blood for up to 2 days, in urine for 10 days, and in hair for 90 days.^4^

Maintenance treatment with buprenorphine or methadone is the current foundation of evidence‐based OUD care because of decades of evidence demonstrating these medications reduce illicit opioid use, and mortality and morbidity. However, both medications do have limitations related to patient compliance, misuse, diversion, risk of overdose and respiratory depression. Only 22% of people with OUD receive medication of any sort. Buprenorphine overdose presents unique problems in that it is not reliably reversed by naloxone due to its long half‐life time and strong binding with receptors. From 2019 to 2021, the 1955 buprenorphine‐related opioid overdose deaths accounted for 2.6% of opioid‐involved overdose deaths, and almost 93% of those deaths included another drug.^5^ Likewise policy changes allowing 28 days of take‐home methadone did not increase methadone‐involved overdose deaths even though overall opioid‐related deaths increased in the setting of fentanyl's continued spread.^6^

Both methadone and buprenorphine have a long half‐life, which is commonly thought to be an advantage for patients as a maintenance therapy. A major complication for such is that there will be more and more opioid users (legal and illegal) in the society as partly reflected in the market needs for therapeutics, which is not what is wanted for the opioid crisis.

In clinical practice, there is a striking similarity between steroids and opioid usage as therapeutics. An escalating dose regimen to reach therapeutic effects is generally used due to the risk of high doses. Sudden stoppage of either opioids or steroid medication after long‐term usage can be life threatening. A taper down strategy is used to stop steroid usage; however, a maintenance therapy approach is taken for opioid usage.

Although there are decades of evidence on the benefits of methadone and buprenorphine maintenance for OUD treatment under well‐defined guidelines, the relapse rate is as high as 30%–70% for current therapies. This indicates that alternative approaches should be explored. The current wisdom of using long‐acting replacement drugs like methadone and buprenorphine is that it allows to maintain a near normal life style without having to break routine to obtain other dangerous illegal opioids or frequent hospital visit. This is different from the debacle of prescription opioid abuse/dependence due to aggressive marketing of long‐acting opioid pain medications, like Oxycontin with the propaganda to bring life back to “normal” with long‐acting potent opioids that were erroneously touted as safe and convenient.

There are many biological changes that occur gradually during chronic opioid and steroid use that lead to dependence. There is a gradual tapering off process where the body is allowed to adjust gradually to decreasing amounts of steroid until the natural balance that existed before drug treatment is regained. This paradigm can and should be tried for opioid dependence among individuals who seek to taper off buprenorphine or methadone after reaching stability in their OUD: the highly complex regulated endogenous opioid system is allowed to slowly adjust from its totally dysfunctional state, during OUD, back to a normal state. This gradual tapering should be achieved with short‐acting opioids that are not or are less addictive, not with drugs like long‐acting methadone or buprenorphine with highly addictive properties.

Some experts have started advocating the use of short‐acting potent opioids, especially in hospital settings, to rapidly mitigate symptoms of pain and withdrawal, at least as a potential bridge for long‐acting methadone or buprenorphine therapy.^7^, ^8^ However, this still does not solve the problem in the large picture because these potent short‐acting alternatives are still strongly addictive.

Addiction, including OUD is a complex problem with physical and psychological dependence, as well as social, psychiatric and motivational issues all playing a part in relapse. Even though maintenance is the foundation of evidence‐based care, there are some patients who prefer opioid‐free recovery and there are others who achieve stability in their OUD and who wish to taper off buprenorphine or methadone. These patients could benefit initially from a gradual opioid tapering using quick onset and short‐acting opioids without known addiction properties. Whereas buprenorphine taper is clinically used, it takes very long time to taper down due to it long half‐life and it is associated with post‐taper overdose death, and buprenorphine itself is addictive.^9^ In the future drug development for addiction therapy, short‐acting regimen for an easy taper down or taper off opioids should be explored. We and others have found that dezocine, a previously FDA approved opioid, is a potential candidate for such purposes (Dalgan, average half‐life 2.2 h).^10^, ^11^, ^12^, ^13^ It was discontinued in the US market around 2011 following its substance matter patent expiration. Unlike other opioids, there are no reports in the English language medical literature of addiction or abuse or associated death with dezocine despite decades of clinical usage. It is a partial mu opioid receptor (MOR) agonist without significant beta‐arrestin pathway activation and is a kappa opioid receptor antagonist.^13^, ^14^ It also inhibits both norepinephrine and serotonin transporter proteins, comparable to nisoxetine, an anti‐depressive medication with analgesia properties.^14^ Depression is one hallmark of opioid withdrawal syndrome, and a potential reason for relapse. The unique pharmacological profile could make dezocine a potential alternative to that would allow for opioid weaning due to it quick onset/offset properties that are similar to fentanyl, but with less of the high addiction propensity of fentanyl. This could be a particularly life changing option for those who are highly motivated to quit mu‐receptor agonist clean. Our collective professional and personal experience, as well as clinical data indicate that withdrawal from these long‐acting opioids can precipitate tragic, life ending overdose. Although there is no well‐established model to test the taper down strategy, our recent data indicate that dezocine reduces oxycodone consumption significantly, which is encouraging.^15^ Further studies to determine if dezocine can reduce fentanyl intake would be clinically relevant due to the current fentanyl addiction epidemic. It is important to note that dezocine is not approved for any clinical usage in the US at the present time. Further study will be required for FDA approval. Since intranasal administration is proposed, a novel safe formulation is warranted. The pharmacokinetics of the intranasal administration and the optimal dose needs to be determined. New toxicology studies will be needed for intranasal administration. There is significant need to find alternatives to bridge buprenorphine therapy for fentanyl addiction. Trials to demonstrate whether dezocine could potentially be an alternative should be considered. In summary, we are proposing to introduce a non‐scheduled short‐acting opioid into clinical practice given the significant need to find alternatives to taper off opioids and bridge buprenorphine and methadone therapies for OUD. Dezocine is a potential candidate to explore.

## CONFLICT OF INTEREST STATEMENT

Dr. Renyu Liu is the founder of the Neurokappa Therapeutics in developing novel therapeutics for neurological diseases including stroke and addiction.

## Data Availability

Data are available upon requests to the corresponding author.
